# Research priorities for homecare for older people: A UK multi‐stakeholder consultation

**DOI:** 10.1111/hsc.13991

**Published:** 2022-09-22

**Authors:** Gareth O'Rourke, Bryony Beresford

**Affiliations:** ^1^ Social Policy Research Unit University of York York UK

**Keywords:** home care, multi‐stakeholder consultation, older people, research priorities

## Abstract

Homecare is generally understood to refer to services that support people to continue living in their own homes. Older people are the primary users and many countries report an increase in the number using homecare services and greater spending on such provision, driven in part by investment in ‘ageing in place’ policies. Despite this, and reflecting social care more generally, homecare is relatively under‐researched. However, in the UK at least, there is growing interest and investment in social care research. In order that this investment is not wasted, it is essential that research addresses what stakeholders identify as research priorities. This study reports work undertaken in the UK during 2021/22 to identify research priorities for homecare for older people, and a broad scoping of existing evidence. A two‐stage consultation process was used. First, topic areas for research were identified through consultations with stakeholders. Second, a survey ascertained agreement and differences between groups regarding the relative importance of topic areas as research priorities. Over 50 people participated including older people (*n* = 7), family members (*n* = 11), homecare workers (*n* = 16), homecare providers (*n* = 9) and national policy, evidence and advocacy leads (*n* = 13). Twenty discrete research topic areas were identified. Only one topic area (*Joint working between homecare and health services*) was a ‘Top 5’ research priority for all stakeholder groups. *Timely engagement with homecare* and *Workforce: recruitment and retention* were ‘Top 5’ priorities for three stakeholder groups. Scoping of existing research indicates that topic areas receiving the most research attention to date are not among those identified as being of high priority for research. To our knowledge, this is the first time research priorities for homecare have been generated. Findings will be of value to research funders, organisations using research evidence and the research community.

**What is known about this topic?**

- Homecare is an increasingly important component of social care for older people but remains relatively under‐researched.
- To avoid ‘research waste’, it is important that future research on homecare responds to the needs and priorities of stakeholder groups.

**What this paper adds?**

- Research priorities for homecare range from micro to macro issues, with stakeholder groups appearing to vary in the topics they would prioritise for research.
- There is evidence that homecare research to date has not attended to the issues which stakeholders prioritise.

## 1 INTRODUCTION

‘Homecare’ is broadly understood to refer to services that allow older people (predominantly) to continue to live in their own homes. Many countries have seen year‐on‐year increases in the number of older people using homecare, driven both by demographic changes and the implementation of ‘ageing in place’ policies (Kristinsdottir et al., 2021; OECD, 2020). However, there is considerable variation between countries in how this is organised and funded, and what tasks and activities are regarded as constituting homecare (Contandriopoulos et al., 2021; Incisive Health, 2018).

In the UK, homecare includes help with personal care (e.g. washing, dressing and toileting), meal preparation and eating and support with essential domestic tasks (Bottery et al., 2018; NICE, 2015). It may also include support to achieve wider goals such as social participation (Jasper et al., 2019). Homecare can take the form of long‐term care, or a shorter‐term intervention to facilitate recovery and reablement (Bennett & Hodge, 2021; Beresford et al., 2019). Although primarily regarded as a social care service, homecare is sometimes coordinated with community health services and can include elements of healthcare (e.g. administering some medications; Hu, 2014; Zimpel‐Leal, 2018). However, UK countries differ in the level of care which is publicly funded, with Scotland, Wales and Northern Ireland spending proportionately more on older people's care than England (Atkins et al., 2021). Across all countries, those who do not have eligible needs, or have financial means above the limit allowed, may purchase homecare privately as ‘self‐funders’.

UK homecare providers are mostly private for‐profit organisations ranging in size and scope from small to medium‐sized local operators to large national and international companies. The sector also includes not‐for‐profit providers of varying size, and some local authorities have ‘in‐house’ homecare services. Over 12,000 providers are registered with regulatory bodies and employ around 585,000 homecare workers. Recent estimates indicate that UK local authorities and the National Health Service (NHS) are purchasing homecare services for just under 610,000 people, most of them older people, at a cost exceeding £2.8b p.a. (excluding Scotland and Northern Ireland). The number of self‐funders is not known but the value of the self‐funding market has been estimated at £1.5b p.a. (Houghton & The Homecare Association, 2020).

Recent growth in demand for homecare in the UK has been driven, in large part, by ageing of the population. This is forecast to increase to one in four people aged 65+ in 2050 compared to one in five in 2019 (ONS, 2021). This increase will be accompanied by a greater number aged 75+ living with multiple long‐term health conditions and associated support needs (Falkingham et al., 2010; Kingston et al., 2018). Alongside this significant demographic change, there is early evidence of increased awareness of, and a preference for, homecare, with the COVID‐19 pandemic likely to have contributed to this (UKHCA, 2020; YouGov, 2021).

Together these factors highlight the critical need for high‐quality evidence on homecare to be available to policy makers, homecare commissioners and providers, frontline staff and older people and their families. Currently, however, such evidence is limited. This is because, overall, social care has not received similar research attention or investment compared to healthcare. Furthermore, within the field of social care for older people, the evidence base for homecare is, arguably, even weaker than that for care homes.

An important first step towards improving the evidence base on any population or issue is to consult with relevant stakeholder groups about what they think are the research priorities (Grill, 2021; Nygaard et al., 2019; Razavi et al., 2020; Staley & Hanley, 2008). This can then be used to guide and inform research efforts and funding. There has been some work on research priorities for populations likely to become homecare users (Bethell et al., 2019; Kelly et al., 2015; Schipper et al., 2014; SPRU, 2020), and wider work on research priorities for adult social care includes homecare (Cyhlarova & Clark, 2019; NICE, 2015, 2021). However, to our knowledge, there has been no work that has specifically sought to identify research priorities for homecare for older people. In the UK, such an endeavour is particularly timely, and needed, given the growing investment by the government in social care research (NIHR, 2018, 2022).

This study reports a multi‐stakeholder UK consultation on research priorities for homecare and a parallel ‘evidence scoping’ exercise. The objective was to identify and specify priority areas for homecare research, report stakeholders' views on their relative importance and scope the existing evidence base. The intended primary audiences were research funders, organisations that use evidence and research data for national strategic/policy work and the research community.

The REPRISE checklist (Tong et al., 2019) informs the content of the paper.

## 2 METHODS

### 2.1 Project objectives

The aim was to identify topics, or issues, regarded as priorities for research from the micro to macro‐level. That is, spanning experiences of care giving and receiving through to service organisation and delivery and to policy making. We were not aiming to achieve a consensus on the relative importance of identified research priorities, or develop a prioritised list of research questions. Rather, the objective was to understand the relative importance to different stakeholder groups of topics identified as research priorities.

The approach taken was informed by arguments for inclusive approaches to research priority setting in healthcare (Pratt et al., 2016; Razavi, 2019; Razavi et al., 2020); as well as accounts of previous research prioritisation projects involving older people (Bethell et al., 2019; Emrich‐Mills et al., 2019; Patel et al., 2021; Schipper et al., 2014).

### 2.2 Project design and team

The project comprised the following components:

- Consultation with key stakeholder groups
  - Stage 1: Parallel consultations to elicit views on research priorities for homecare.
  - Stage 2: Survey to collect consultees' views on the relative importance of topics identified as research priorities.
- Targeted searches of research databases to identify the extent of existing research relevant to the topic areas identified as research priorities. This comprised searches for relevant systematic reviews and UK‐based studies only.

Two applied health and care services researchers worked on the project, both with extensive experience in consultation work with different types of the stakeholder group, and one (BB) with previous experience in multi‐stakeholder research prioritisation work (Beresford et al., 2017; Booth et al., 2018).

### 2.3 Stakeholder groups consulted

We consulted the following groups:

- Older people currently using homecare (homecare users).
- Older people who may, in the future, need or choose to use homecare (potential homecare users).
- Family members of older people using homecare, including those providing unpaid care (family members/carers).
- Homecare workers.
- Senior professionals: in homecare business leadership and management roles; and/or strategic development or policy advocacy roles.

### 2.4 Ethical considerations

Ethical approvals are not required for research prioritisation projects (Health Research Authority, 2017).

### 2.5 Recruitment

Four homecare providers, known to the research team through a pre‐existing research collaboration, distributed the project invitation (via post or email) to a small number of selected clients, family members/carers and homecare workers. All predominately served the self‐funder market. All provided a range of different types of homecare (e.g. personal care, dementia care and live‐in care). Invitations were also distributed to individuals (family members/carers and potential homecare users) who had previously contacted the project team expressing an interest in contributing to the team's wider programme of work on homecare. ‘Senior professionals’ included those already known to the project team, or research colleagues, and others identified via desk‐based research. They were invited to take part via email.

An information leaflet about the project (different versions for each stakeholder group), and a data privacy notice, accompanied the invitation.

### 2.6 Stage 1 consultation

In designing the project, we were conscious of the potential for some voices to be heard over others (Nygaard et al., 2019), and the need to be flexible in how we consulted with the different stakeholder groups (Abma & Broerse, 2010; Madden & Morley, 2016; Patel et al., 2021; Pollock et al., 2014), especially at a time of continuing Covid‐19 restrictions on face‐to‐face meetings. Methods used therefore responded to stakeholder groups' needs and preferences and, aside from senior professionals, multiple ways of participation were offered, see Table 1.

**TABLE 1:** Stage 1 consultees and consultation methods

Stakeholder group	*N*	Consultation methods
Older people: *homecare users*	3	Individual telephone interviews. (25–40 min; *n* = 3)
Older people: *potential homecare users*	4	Online focus group ×1 (90 min; *n* = 3) Written response (*n* = 1)
Family members/carers	11	Online focus group ×1 (60 min; *n* = 4) Individual telephone interviews (30–70 min; *n* = 6) Written response (*n* = 1)
Homecare workers	16	Online focus group ×2 (90 min; *n* = 13) Individual telephone interviews (25–35 min; *n* = 3)
Senior professionals: *Homecare providers* (*9*) *Third‐Sector organisations* (*3*) *National advisers* (*2*) *Government policy/research roles* (*5*) *LA commissioning/assessment* (*3*)	22	Online workshop ×1 (3 h)

All consultees received materials in advance to help them prepare for the discussion. Other than senior professionals, this took the form of a short booklet with different versions produced for different stakeholder groups (for an example, see Supplementary File: SuppInfo_1_OP_booklet.pdf), which briefly described the project objectives, set out the areas for discussion and provided space to make notes in advance if wished. A week prior to the workshop for senior professionals, participants were invited to share views on research priorities for homecare via a short online survey hosted by Qualtrics (Qualtrics Provo UT, 2022). It was made clear to all that preparatory activity was not compulsory.

Drawing on the approach taken in other research prioritisation frameworks (Abma & Broerse, 2010; Grill, 2021; JLA, 2021), the basic structure of all consultations was the same. Thus, two contexts/experience‐sharing discussions preceded a discussion about research priorities (See Supplementary File: SuppInfo_2_consultation_topics.pdf). Topic guides ensured comprehensive coverage and consistency across consultation encounters. Group discussions were facilitated by both members of the project team. Telephone interviews were carried out by GOR.

Those taking part, other than senior professionals, received a £25 ‘thank you’ gift voucher.

### 2.7 Data analysis

Focus groups and telephone interviews were audio recorded and an inductive approach was taken to data analysis. First, two sets of data were extracted from the recordings: (i) research questions directly verbalised by consultees, and (ii) descriptions of problematic experiences or concerns. These data were extracted into excel spreadsheets (organised according to stakeholder group) through a process of repeated listening to the recordings. Problematic experiences or concerns were then ‘translated’ into the form of research questions by the research team. Data from written submissions were treated similarly. Each question was given a code identifying the stakeholder group that suggested it. Questions were then sorted and grouped into discrete (non‐overlapping) topic areas through an iterative process involving both members of the project team. Each topic area was given a title followed by a short ‘definition’, which summarised all questions contained within it.

### 2.8 Stage 2 consultation

A survey was used to collect consultees' views about the relative importance of the research priority topic areas identified in Stage 1. The main objective was to explore whether there were differences between stakeholder groups in terms of topic areas regarded as high and low priority for research.

First, consultees were asked to select the five topic areas they considered to be the highest priority for research, and then to rank them from 1 to 5 (1 = most important, 2 = second most important, etc.). Consultees were then asked to select the five topic areas they considered to be the lowest priority for research. A ‘free text’ box was offered at the end of the survey for comments. A plain English version of the survey was used for all groups other than senior professionals.

For senior professionals, an online survey hosted by Qualtrics (Qualtrics Provo UT, 2022) was used. All other groups were offered the option of completing a postal (see Supplementary File: SuppInfo_3_consultation_survey.pdf) or online version (where the email address was available). In all instances, the survey was accompanied by a two‐page document setting out the research priority topic areas and short definitions. Homecare users were telephoned by a member of the project team to offer help if required. Two reminders were used with senior professionals, and one for the other groups. Those completing the survey, other than senior professionals, received a £20 ‘thank you’ gift voucher.

### 2.9 Data analysis

Survey data were downloaded or entered into Excel and analysed using descriptive statistics and sorting functions. The number of high‐priority votes cast for each topic area was calculated to determine the ‘Top 5’ topic areas for each stakeholder group. Where two or more topic areas received the same number of votes, the combined 1–5 rankings of consultees in that stakeholder group were used to order them. Topic areas receiving no Top 5 votes from a stakeholder group were identified. The number of lowest priority votes cast for each topic area was calculated for each stakeholder group. Comments were coded and grouped according to content.

### 2.10 Scoping existing evidence

Focused searches were undertaken to identify systematic reviews and UK‐based studies relevant to the topic areas identified, including work in progress. We used the existence of systematic reviews as an indicator of research interest in a topic and the number of included studies as an indicator of the extent of the existing evidence base.

PROSPERO and Cochrane databases were searched for systematic reviews. The National Institute for Health Research (NIHR) Journals Library and the United Kingdom Research and Innovation (UKRI) gateway portal were searched for current or completed UK‐based studies. The search terms: ‘home care’, ‘homecare’, ‘domiciliary care’, ‘social care’, ‘older’ and ‘older people’ were used in different combinations according to the search facility of each database. Searches took place between the 10th and 14th of December 2021. In addition, a manual search of NIHR's School for Social Care Research (SSCR) website's listing of currently funded and completed studies was undertaken on 15th December 2021.

Inclusion criteria were as follows:

- study concerned homecare (all or some) provided as social care;
- the study population was, or included, older people (65+ years) and
- research objective(s) relevant to one or more of the identified research priority topic areas.

Systematic reviews were excluded if they were registered before December 2017 and no output paper was recorded or found via a Google Scholar search of the review title. UKRI Innovate projects with no research element and ESRC awards for PhD studentships were excluded. Screening of titles/abstracts was undertaken by one member of the research team (GOR) who consulted with another (BB) when uncertain. UK studies identified that were included in any of the systematic reviews identified were excluded. Where systematic reviews and studies were relevant to more than one topic area, they were assigned to the main topic area they addressed. For example, we used the primary outcome to allocate reviews on effectiveness.

## 3 FINDINGS

### 3.1 Stage 1 consultation

Details of the sample (*n* = 56) are set out in Table 1. For most groups, the approach taken to sharing invitations means we do not have information on take‐up. However, for senior professionals, of the 41 approached (or, infrequently, the person they nominated), 31 signed up for the workshop and 22 attended.

Analysis of consultation discussions identified 179 questions considered to be amenable to research. Of these, 42 were suggested by older people, 40 by family members/carers, 42 by homecare workers and 55 by senior professionals. These were sorted into discrete topic areas (*n* = 20), see Table 2. A total of 15 of the 20 topic areas emerged from research questions generated from consultation work with at least two stakeholder groups. Of these, four topic areas were raised by all groups. These were as follows: *Mapping & understanding the homecare population, providers, and workforce*; *Timely engagement with homecare*; *Understanding homecare as a relationship‐based intervention*; and *Workforce: recruitment & retention*. Five topic areas emerged from discussions with just one stakeholder group and are identified by a triangle in Table 2.

**TABLE 2:** Topic areas: Titles and definitions

Topic area title and definition	
**Understanding, defining and measuring the components of homecare and homecare outcomes**. This concerns research that would: (i) identify and define the components, or active ingredients, of homecare associated with user outcomes and experiences; and (ii) identify and agree on the outcomes which evaluative research should include (i.e. core outcomes set)	
**Mapping & understanding the homecare population, providers and workforce**. This topic concerns research to describe the homecare population and their needs, the types of organisations providing homecare and the workforce, including skills and capabilities	
**Public sector funding of homecare**. This is about the cost‐effectiveness of greater public investment in homecare	
**Homecare compared to other care options**. This is about whether the type of care (e.g. homecare vs. residential care vs. supported care) affects outcomes and experiences. It also includes people's preferences with respect to the place of care	
**Timely engagement with homecare**. This includes questions about whether there are reliable predictors (or early warning signs) of the need for social care, the effectiveness and cost‐effectiveness of ‘pre‐emptive’ or early use of homecare and how to shift public understanding of homecare	
**Navigating and decision‐making about homecare**. This concerns research to address older people's and family members' desire for standardised information on homecare worker quality and identifies how to best support informed decision‐making about the choice of provider and paying for care	
**▲Homecare as a preventive health intervention**. This topic concerns work to investigate the association between the use of homecare and use of healthcare services, and whether the delivery model affects this	
**Understanding and comparing the different models of homecare in terms of outcomes, resource use/costs, process and experience**. This topic is about homecare delivery models: how they differ in what they offer and the way they work, and how they compare in terms of outcomes and experience (user, family member and homecare worker) and relative cost‐effectiveness	
**Integrating an enabling approach into homecare**. This topic is about whether an enabling approach (or reablement) should be integral to homecare practice	
**Understanding homecare as a relationship‐based intervention**. This topic is concerned with understanding homecare as an intervention based on relationships. It covers issues such as the need to better understand the user–homecare worker relationship; how the home setting influences that relationship and the way it is experienced and managed; and how the quality of the relationship impacts users' and workers' experiences and outcomes	
**Homecare as a social intervention**. This topic is about homecare as a social intervention and its potential impact on users' social connectedness, and whether this varies between delivery models	
**▲Joint working between homecare and healthcare services**. This topic concerns joint working between homecare and healthcare services, including what needs to be in place to ensure such arrangements are effective, including cost‐effective	
**Family involvement in homecare**. This topic is about the relationship among homecare workers, care recipients and family members, and factors that impede or support positive relationships which benefit all parties. It also includes the issue of shared electronic records as a tool to support family involvement	
**Housing and homecare**. This topic concerns the role of housing factors (e.g. physical layout, location and housing quality) on access to, and delivery and outcomes of, homecare. It also includes the notion of ‘future‐proofing’ housing decision‐making amongst younger older people and how receiving homecare may affect people's feelings about their homes	
**Workforce: recruitment and retention**. This topic covers the identification of strategies and practices which would support recruiting and retaining high‐quality homecare workers	
**Workforce: supervision, support and training**. This topic concerns mapping and evaluating current training and supervision practices and identification of ways in which this could be improved. Also included are the issues of peer support and peer learning	
**Technology: supporting the delivery of care**. This topic is about the digitisation of care records and its impacts on the quality and safety of care	
**▲Technology: meeting care needs**. This topic is about the use of technology to meet care needs or augment hands‐on care. This includes evaluating current technologies and horizon scanning for promising technologies, including existing technologies which have the potential to translate into a homecare application	
**▲Managing complaints about homecare**. The focus of this topic is the experience and outcomes of families who have concerns and/or have raised a complaint about a homecare service, and the adequacy of current processes and systems which respond to concerns and complaints	
**▲Person‐centred needs assessment**. This topic is about the types of assessment practices used by local authorities and NHS organisations to determine eligibility for homecare in relation to needs, and the extent to which processes of needs assessment are truly person centred	
**Key**:	▲ = Topic areas suggested by one stakeholder group only

### 3.2 Stage 2 consultation

Three of the senior professionals who took part in Stage 1 were no longer in post or available at Stage 2, yielding a total potential survey sample of 53 consultees. Forty‐one completed the survey (77% response rate). The response from each stakeholder group was as follows: 4/7 older people; 9/11 family member carers; 13/16 homecare workers; and 14/19 senior professionals. One respondent did not identify which stakeholder group they represented, and their data could not, therefore, be included in the analysis. Four further respondents did not provide complete data on the topic areas they felt were of the lowest priority for research. We note that seven respondents commented that they found it difficult, or were unwilling, to identify topic areas as being of the lowest priority because they felt all were important and/or interconnected. This goes some way to explaining incomplete responses to this survey item.

Findings are summarised in Table 3. One topic area was a ‘Top 5’ research priority for all stakeholder groups and two more were a ‘Top 5’ research priority for three groups. These were as follows: *Joint working between homecare and healthcare services*; *Timely engagement with homecare*; and *Workforce: recruitment and retention*. One topic area (*Technology: supporting delivery of care*) did not receive any high‐priority votes from three of the four stakeholder groups. The number of ‘lowest priority’ votes for the topic area by stakeholder group is shown in Supplementary File: SuppInfo_4_lowest_priority_votes.pdf. Overall, these support findings across stakeholder groups regarding the topic areas of highest priority, with no (or very few) ‘lowest priority’ votes received for *Joint working between homecare and healthcare services; Timely engagement with homecare*; and *Workforce: recruitment and retention*.

**TABLE 3:** Stakeholder group priorities (*n* = 40)

Topic area		OP	FMC	HCW	SP
Understanding, defining and measuring the components of homecare and homecare outcomes				✓	
Mapping and understanding the homecare population, providers and workforce		✘	✘		✓
Public sector funding of homecare		✓			✓
Homecare compared to other care options		✘	✘		
●Timely engagement with homecare		✓	✓	✓	
Navigating and decision‐making about homecare			✓	✘	
▲Homecare as a preventive health intervention					✓
Understanding and comparing the different models of homecare …….				✓	
Integrating an enabling approach into homecare		✓			
Understanding homecare as a relationship‐based intervention		✘			
Homecare as a social intervention		✓			
●▲Joint working between homecare and healthcare services		✓	✓	✓	✓
Family involvement in homecare		✘			
Housing and homecare				✘	
●Workforce: recruitment and retention			✓	✓	✓
Workforce: supervision, support and training		✘			
Technology: supporting the delivery of care		✘	✘	✘	
▲Technology: meeting care needs					
▲Managing complaints about homecare					✘
▲Person‐centred needs assessment			✓		
Key:	✓ The five topic areas receiving most ‘highest priority’ votes	✘Topic areas receiving no ‘highest priority’ votes			
	● = Topic areas that three or more stakeholder groups voted a Top 5 priority				
	▲ = Topic areas generated by one stakeholder group only				

As with voting for high‐priority areas, there were also differences between stakeholder groups in terms of what emerged as low‐priority topic areas. Sometimes the split of opinion was between older people and family members/carers versus homecare workers and senior professionals. For example, *Integrating an enabling approach into homecare*; *Understanding homecare as a relationship‐based intervention* and *Person‐centred needs assessment* were never identified as low‐priority topics by older people or family members/carers, but were by at least some respondents from the other stakeholder groups. In other cases, the split of opinion was between senior professionals and the other stakeholder groups. For example, only one senior professional considered *Homecare compared to other care options* as low priority, yet at least half of the members of other stakeholder groups regarded it as such.

### 3.3 Scoping the existing evidence

**FIGURE 1:**
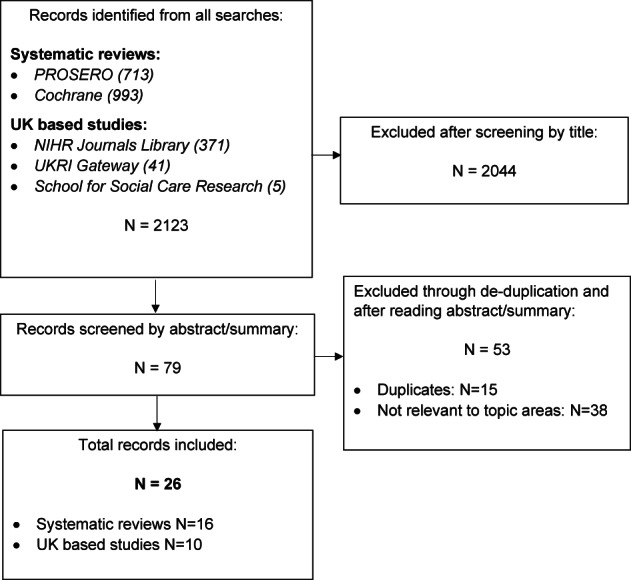
PRISMA diagram.

Figure 1 summarises the outputs of searches and the screening process.

A total of 16 systematic reviews (Backhouse et al., 2021; Bennett & Hodge, 2021; Cochrane et al., 2016; Crocker et al., 2021; Cunningham et al., 2020; Dawson et al., 2020; Lucien et al., 2020; Malden et al., 2021; McGill, Malden, Alex, et al., 2021; McGill, Malden, Frost, et al., 2021; Montgomery et al., 2008; Ogwu et al., 2020; Spiers et al., 2019; Szczepura et al., 2020; Whitehead et al., 2015; Young et al., 2017) (seven completed and nine ongoing) relevant to research priority topic areas were identified (PROSPERO = 13; Cochrane = 3). Of these, two were reviews of systematic reviews (Cunningham et al., 2020; Dawson et al., 2020). The earliest review included was completed in 2008 (Montgomery et al., 2008). In addition, 10 UK‐based studies (NIHR HSDR, 2017a, 2017b; NIHR SSCR, 2013, 2018, 2019, 2020, 2021; UKRI, 2015, 2020, 2021) (six completed and four ongoing) were identified (NIHR Journals Library = 2; UKRI gateway = 3; and NIHR SSCR website = 5). The earliest study included was completed in 2013 (NIHR SSCR, 2013). Details of the systematic reviews are provided in Supplementary File: SuppInfo_5_systematic_reviews.pdf and the UK‐based studies in Supplementary File: SuppInfo_6_UK_based_studies.pdf.

Table 4 reports the number of systematic reviews (SR) and studies identified as relevant to each topic area. Clusters of research activity (defined as at least two systematic reviews) were identified for three topic areas, as follows:

- *Homecare compared to other care*:
  - two completed reviews: total studies included = 14
  - one ongoing review
- *Integrating an enabling approach into homecare:*
  - two completed reviews: total studies included = 15
  - two ongoing reviews
  - one completed study
- *Technology: meeting care needs*
  - three ongoing systematic reviews.

**TABLE 4:** Number of identified systematic reviews and UK‐based studies per topic area

Topic area	Systematic reviews		UK based studies	
	Completed	On‐going	Completed	On‐going
Understanding, defining & measuring the components of homecare and homecare outcomes				
Mapping & understanding the homecare population, providers, and workforce		2		1
Public sector funding of homecare				
Homecare compared to other care options	2	1		
●Timely engagement with homecare				
Navigating & decision‐making about homecare			2	1
Homecare as a preventive health intervention	2			
Understanding and comparing the different models of homecare …….			2	
Integrating an enabling approach into homecare	2	2	1	
Understanding homecare as a relationship‐based intervention				1
Homecare as a social intervention		1		
●Joint working between homecare and health care services				
Family involvement in homecare				
Housing and homecare				1
●Workforce: recruitment & retention				
Workforce: supervision, support and training	1		1	
Technology: supporting the delivery of care				
Technology: meeting care needs		3		
Managing complaints about homecare				
Person‐centred needs assessment				
**Key**: ● = Topic areas that three or more stakeholder groups voted a Top 5 priority				

For eight of the remaining topic areas, at least one systematic review and/or UK‐based study was identified. For nine topic areas, no existing or current research was identified.

## 4 DISCUSSION

To our knowledge, this study reports the first piece of work to identify research priorities for homecare for older people, ranging from micro to macro‐issues. Aware that research prioritisation work can lead to some voices being heard over others (Nygaard et al., 2019), we did not seek to achieve cross‐group consensus on research priorities. Furthermore, for the groups likely to be less familiar with taking part in research or group/workshop events (older people, family members/carers and homecare workers), we adopted a flexible approach, offering multiple ways to take part. (Abma & Broerse, 2010; Madden & Morley, 2016; Patel et al., 2021; Pollock et al., 2014). We also used materials that enabled those involved to prepare, or feel ready for, the consultation encounter.

This approach proved highly successful. It was evident that many of our consultees had used the pre‐consultation materials to prepare. Lively and insightful discussions produced rich data and, crucially, generated a wide range and a large number of research questions. Each stakeholder group contributed a similar number of questions relative to their size. The high response rate to the Stage 2 survey was indicative of strong engagement in the project.

It was possible to organise the research questions generated from this consultation work into 20 discrete topic areas. This was not contrived in any way as a neat or manageable number: it is simply the number that emerged from our analysis. The fact that each stakeholder group uniquely contributed one or more topic areas (see Table 2) further reinforces the importance of a multi‐stakeholder approach to research priority setting and the need to overcome barriers to participation, particularly for ‘user’ and ‘citizen’ groups (Madden & Morley, 2016; Yoshida, 2016).

The broad scope of topic areas reflects the project's objective to identify research priorities from the micro through to the macro and illustrates the complex nature of homecare and associated evidence, or knowledge, and needs, especially when considered from the different viewpoints of our stakeholders. This calls attention to the need for a broad programme of multi‐disciplinary research drawing on a wide range of study designs and methods.

Two of the identified topic areas (*Understanding, defining and measuring the components of homecare and homecare outcomes; and Mapping & understanding the homecare population, providers, and workforce*) can be considered as ‘foundational’ in that they will generate evidence or outputs which will support, or allow, robust research in other topic areas. In some cases (e.g. outcome measurement and specification of the components of homecare), addressing these topics would also support consistency across studies and, therefore, greater opportunities for synthesis of findings. However, these two topic areas did not feature among those identified as a higher priority by all or most of the stakeholder groups. It is possible that this was because they do not appear to relate directly to key issues and concerns identified by many of our consultees. This may present something of a dilemma for research funders and the research community, conscious of the need to maximise the effectiveness of research and unblock barriers to research, at the same time as being responsive to felt knowledge needs.

Three topic areas emerged as high priorities for research for all or three of four stakeholder groups. The fact that, across all stakeholder groups, they were rarely nominated as low‐priority topics further supports the perception of shared concerns and perceived knowledge needs related to these topic areas. We briefly consider each in turn.

*Joint working between homecare and healthcare services* was the only topic area voted a Top 5 priority by all stakeholder groups reflecting, perhaps, strong views on the need for improved joint working and the potential impacts this may have. As a topic, it aligns well with policy and practice initiatives in pursuit of ‘integrated’ care (Baxter et al., 2018; Briggs et al., 2018), arising from difficulties of coordinating health and social care organisations to operate collectively as a single ‘system’ (Edmonstone, 2020). Evidence indicates that this may result in poor communication between health and homecare providers (Hestevik et al., 2019).

*Timely engagement with homecare* covers issues such as the identification of predictors of the need for social care, effectiveness/cost‐effectiveness of ‘pre‐emptive’ homecare and public understanding of homecare. This aligns well with the recent emphasis in social care policy on prevention (Department for Health and Social Care, 2022; Hernandez & Wittenberg, 2014; SCIE, 2021; Skills for Care, 2019). However, it must also be understood in the context of differing understandings of what constitutes prevention (Marczak et al., 2019; Verity et al., 2022), as well as reduced public spending on low‐intensity social care services and more stringent needs assessments for access to local authority‐funded services (Thorlby et al., 2018).

*Workforce: recruitment and retention* is, perhaps, not unexpected as a priority given longstanding workforce pressures in the sector. These have been exacerbated over the short/medium term by the Covid‐19 pandemic (Turnpenny & Hussein, 2020) and are set to increase over the long term due to continuing ageing of the population (Wittenberg et al., 2018). The poor status and rewards offered to the homecare workforce are frequently cited as major contributory factors (Care Quality Commission, 2021; Homecare Association, 2021; Timonen & Lolich, 2019). Within this topic area, research questions identified by stakeholders included calls for detailed evidence of what attracts people to homecare work and the factors that cause them to remain or to leave. Homecare as a ‘profession’ was a key concern in terms of values, knowledge base and competencies of homecare workers, as well as their standing in relation to the colleagues that work alongside them in ‘regulated’ professions.

The differing outcomes of voting for lowest priority topic areas across stakeholder groups also illustrate the particularity of viewpoints within homecare and further support our decision not to employ a process that would force a consensus across stakeholder groups. Only one topic area (*Technology: supporting the delivery of care*) was considered to be a low priority by most stakeholder groups, the exception being senior professionals. Research questions falling under this topic concerned evaluating the impact of electronic rostering systems and electronic care records on quality and safety. Senior professionals' relatively stronger support for this topic may reflect the fact that, in the UK, there is an expectation that, by spring 2024, all social care providers will be using digital care records (Digital Social Care, 2022; Skinner & Hessey, 2021). Certainly, such a fundamental change in the delivery of social care should not go without careful evaluation. Indeed, existing research suggests the need to be aware of potential disadvantages (Moore & Hayes, 2017, 2018), and difficulties of realising potential benefits in implementation (Renyi et al., 2020; Rocha et al., 2015).

The second component of the project was to scope existing evidence and current UK studies relevant to the topic areas identified. We took a pragmatic approach, focusing only on identifying registered systematic reviews and UK‐based studies. It should therefore be taken only as a starting point but does, we hope, offer a useful resource to the research community in the UK, and gives some sense of the current state of the evidence base on homecare.

Here, there are perhaps three key points to note. First is the overall impression of a relatively limited body of research evidence on homecare, much of which is ongoing. Second, it would also appear that, to date, the bulk of research activity is restricted to a small number of topic areas, with more limited work in some other topic areas. However, we did not identify any systematic reviews or UK‐based studies relevant to 9 of our 20 topic areas.

Third, we found that topic areas that have received the most research attention were not among those identified by our stakeholders as being of high priority for research. Thus, none of the three topic areas with apparently more substantive evidence bases emerged as high priorities for research. Furthermore, we did not identify *any* research relevant to the three topic areas identified as a high priority for research by all or most stakeholder groups. Some caution must be exercised in relation to this finding as some reviews/studies may contribute to other topic areas beyond their primary focus. Nevertheless, this highlights the importance of stakeholder consultation, and research priority setting more generally, as a means of informing research funding and effort and reducing research waste (Boaz et al., 2018; Chalmers et al., 2014; Grill, 2021).

## 5 LIMITATIONS

The work was broad in scope and underpinned by effective consultation with a wide range of stakeholders. However, some limitations should be noted. For pragmatic reasons, recruitment of homecare users, most homecare workers and some family members/carers was undertaken through their homecare provider/employer who predominately provided services to ‘self‐funders’. Furthermore, it was not possible to purposively recruit so as to ensure the inclusion of people whose experience of homecare may have been affected by issues of identity, for example, race, ethnicity, sexual orientation and gender. Only one of the senior professionals directly represented the housing and homecare interface. Finally, our scoping of the literature was limited to systematic reviews registered on PROSPERO and Cochrane and UK studies funded by the National Institute for Health Research and the UK's national research councils.

## 6 CONCLUSION

As far as we are aware, this is the first piece of work identifying research priorities for homecare for older people. Conducted in the UK, consultation work with key stakeholder groups identified 20 topic areas for research, ranging from macro (e.g. mapping the population and delivery models) to micro (e.g. complaints processes) issues. Just three topic areas were a ‘Top 5’ research priority for most or all stakeholder groups. These were as follows: *Joint working between homecare and healthcare services*; *Timely engagement with homecare*; and *Workforce: recruitment and retention*. A ‘first look’ at the existing evidence base indicates that, to date, research activity has not focused on the topics which stakeholders prioritise.

As with all research priority work, the topic areas (or research questions) identified will, to some extent, reflect the policy, service and socio‐demographic context in which the work was conducted, in this instance the UK. This naturally affects the extent to which the findings are transferrable to other countries. This is something that researchers and stakeholders in other countries will need to judge. However, we do believe that, at least, this work can be used as a stimulus or starting point for similar endeavours elsewhere.

Finally, rather than using a standard consultation method, we individualised our methods to each stakeholder group. We believe this approach worked well and secured a strong engagement with the project. We deliberately chose not to seek a consensus on research priorities and instead explored agreements and differences between stakeholder groups. We would argue that both are critical to informing research agendas and research strategies, and to ensure that all voices are heard.

## AUTHOR CONTRIBUTIONS

GOR and BB designed and implemented the consultation and evidence scoping exercise, undertook the data analysis and co‐authored the paper.

## CONFLICT OF INTEREST

The University of York/Home Instead partnership agreement stipulates the academic independence of the university in conducting all research and related activities. Home Instead was not involved in any aspect of the design of the consultation or its interpretation.

## Supporting information


Data S1

Data S2

Data S3

Data S4

Data S5

Data S6

## Data Availability

The data that supports the findings of this study are available in the supplementary material of this article.
