# Supplementary material for: Research priorities for homecare for older people: A UK multi‐stakeholder consultation
Source: Health Soc Care Community. 2022 Sep 22;30(6):e5647–60. doi: 10.1111/hsc.13991 (PMC10087309; doi:10.1111/hsc.13991)
Supplement: Supplementary file 1 — Data S1 [file HSC-30-e5647-s004.pdf]

What do we need to know about home care?

Meeting Information Booklet

We'd like to invite you to a meeting to discuss 'What do we need to know about home care?'. This is one of a series of meetings and conversations we're having with older people, families, home care workers and people who organise or provide home care.

This booklet explains the sorts of things we'd like to discuss at the meeting. If you'd like to, there is space for you to make notes in advance and during the meeting.

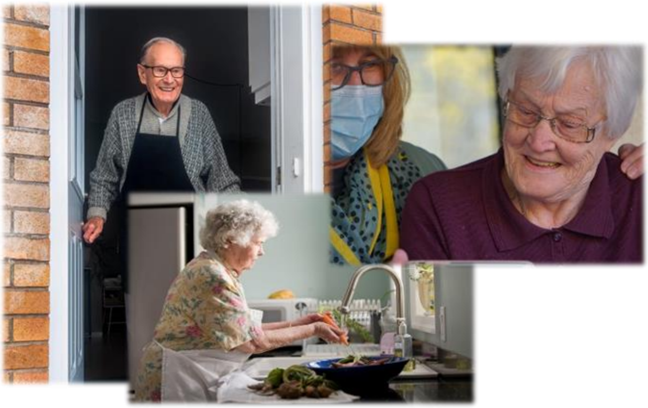

About us

We are social scientists based in the University of York's Social Policy Research Unit (SPRU). SPRU has done lots of research about the well-being, care and support of people with social care needs. You can visit the SPRU website to find out more about the research we do.

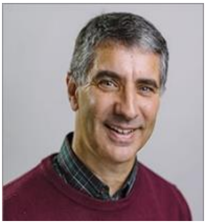

Gareth O'Rourke

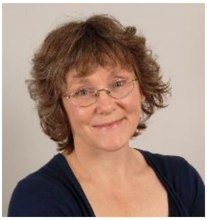

Bryony Beresford

We are currently working on a three-year programme to increase the quantity and quality of research about home care.

Why are we holding these meetings?

Home care affects the lives of very many people but there is not very much research evidence about how best to provide it. It's important that research is focussed on the right things. Therefore, we want to talk to older people who have experience of using home care, as well as those who have not, to ask their views about the most important topics/issues to be researched.

You don't need to know anything about research to take part. We're interested in your experience of home care and/or views about the possibility of using home care in the future.

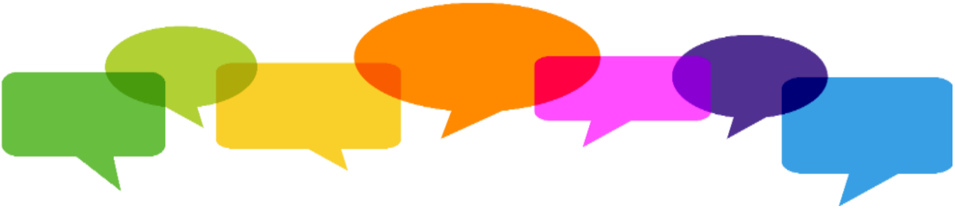

Preparing for the meeting

There are three things we'd like to discuss during the meeting. You might want to take a bit of time thinking about them beforehand. We've provided some spaces if you would like to make a few notes.

Discussion 1: Choosing and arranging home care

We'd like to hear about your experiences or thoughts about the process of choosing and arranging home care for yourself or a family member. What things helped with making the decision and arrangements; or do you anticipate would be helpful to you in that situation? Similarly, what things made it more difficult, or would you find unhelpful? What needs to change in the way people understand and are making decisions about home care?

Discussion 2: Using home care

We'd like to hear about your experiences or expectations of home care for yourself or a family member. What differences has it made to your lives, or do you hope it will make? This may be actual or anticipated improvements, or things you have found/would find difficult. What needs to change in the way home care is currently provided?

Discussion 3: What do we need to know more about, or understand better?

Imagine you have £50,000 to spend on research on home care! What topic or issue would you chose to spend your money on and why? You might have a firm idea about this before the meeting, or you might prefer to listen to discussions before deciding.
