# Supplementary material for: Research priorities for homecare for older people: A UK multi‐stakeholder consultation
Source: Health Soc Care Community. 2022 Sep 22;30(6):e5647–60. doi: 10.1111/hsc.13991 (PMC10087309; doi:10.1111/hsc.13991)
Supplement: Supplementary file 2 — Data S2 [file HSC-30-e5647-s005.pdf]

Supporting Information 2. Stage 1 consultation discussion topics.

Stakeholder group	Discussion topics
Older people: homecare users	1. Experiences of deciding to use homecare:<br>2. Experiences of using homecare:<br>3. Views about research priorities for homecare*
Older people: potential homecare users	1. Experiences of homecare through friends or family members, and views about future decisions about homecare:<br>2. Hopes and aspirations for the future, and views and concerns about current care options:<br>3. Views about research priorities for homecare*
Family members/carers	1. Experiences of choosing and arranging homecare:<br>2. Experiences through their family member/cared for person using homecare:<br>3. Views about research priorities for homecare*
Homecare workers	1. Experiences of being a homecare worker:<br>2. Views about aspects of role or practice where uncertainties or information needs:<br>3. Views about research priorities for homecare*
Senior professionals	1. Views on key challenges and concerns in relation to the care of older people.<br>2. Mapping the knowledge gaps: what don't we know about homecare, and why we need to know it:<br>3. Views on the research priorities for homecare**
**Key:**	* = Discussion stimulus question: "*Imagine you have £50,000 to spend on research on homecare. What topic or issue would you spend your money on and why*?"
	** Discussion stimulus question: "*What is your burning question for homecare?*"
