# Supplementary material for: Research priorities for homecare for older people: A UK multi‐stakeholder consultation
Source: Health Soc Care Community. 2022 Sep 22;30(6):e5647–60. doi: 10.1111/hsc.13991 (PMC10087309; doi:10.1111/hsc.13991)
Supplement: Supplementary file 3 — Data S3 [file HSC-30-e5647-s006.pdf]

What do we need to know about homecare?

Your views about research priorities

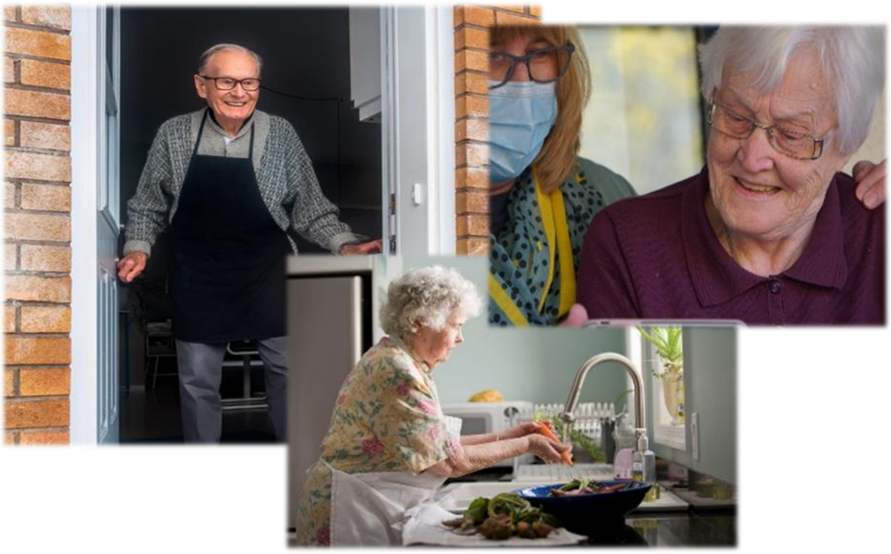

Please complete this booklet to give us your views about priorities for research in homecare.

Please read through all the questions first and then follow the instructions overleaf. You can look at the definitions sheet that came with this booklet for a fuller description of each question.

Please enter your name in the box below before returning your completed booklet in the prepaid envelope provided.

Name:

Thank you

Listed below are the 20 questions about homecare which people have told us we need answers to.

- **First of all**, tick the box by the five questions you think should be the highest priority for research. Place your tick ✓ in the 'My top 5 questions' column.
- **Next**, rank your five top questions in order of importance from 1 to 5, with 1 = most important, 2= second most important etc. Write your ranking next to your tick in the 'My top 5 questions' column e.g. ✓**1**, ✓**2** etc
- **Last of all**, please tell us which 5 questions you think should be given the lowest priority for research. Place a cross ✗ against them in the 'My least important questions' column.

My **top 5** questions✓	Research question	My 5 **least important** questions✗
	1. How does homecare make a difference to people's lives, and how do we measure the difference it makes?	
	2. What sorts of people are receiving homecare, what sorts of organisations provide homecare, and what sorts of people are homecare workers?	
	3. Should the government contribute more to the cost of homecare?	
	4. How does homecare compare to residential care or other care options?	
	5. What can we do to ensure people start using homecare before they reach a crisis because of their health or care needs?	
	6. Is it possible to measure how good a homecare worker is? And how can we make sure people have all the information they need to make the right decisions about homecare?	
	7. Does using homecare affect older people's use of the NHS, or their health care needs?	
	8. Does the type of homecare someone gets make a difference to whether their care needs are met, and how happy they are with their care? Are some types of homecare better value for money than others?	

My **top 5** questions✓	Research question	My 5 **least important** questions✗
	9. Should homecare always try to help older people regain or retain at least some independence?	
	10. What are the relationships between homecare workers and older people like? What helps to make sure these are good relationships for everyone?	
	11. Should we think about homecare as something which helps prevent social isolation and loneliness?	
	12. How can we make sure homecare services and the NHS (including GPs) work together in ways that best meet older people's needs?	
	13. How can we support positive and helpful relationships between homecare workers and family members, and make sure that family members are kept informed and involved?	
	14. How does someone's housing (e.g. location, layout, room size, condition) affect the way homecare is provided to them, and the difference it makes?	
	15. How can we make sure there are enough high-quality homecare workers?	
	16. What are the best ways to train and support homecare workers?	
	17. Does using computer-based care records (rather than paper) make care better and safer?	
	18. How might technology help meet people's care needs at home?	
	19. How can we make sure complaints about homecare are properly dealt with?	
	20. How are older people's homecare needs assessed, and what needs to be improved?	

Office use only:	
Consultee code	
