# Supplementary material for: Research priorities for homecare for older people: A UK multi‐stakeholder consultation
Source: Health Soc Care Community. 2022 Sep 22;30(6):e5647–60. doi: 10.1111/hsc.13991 (PMC10087309; doi:10.1111/hsc.13991)
Supplement: Supplementary file 4 — Data S4 [file HSC-30-e5647-s001.pdf]

Supporting Information 4. Lowest priority votes for each topic area by stakeholder group

**Topic area**	**OP** N=4	**FMC** N=8	**HCW** N=11	**SP** N=13
Understanding, defining and measuring the components of homecare and homecare outcomes.	0	2	2	6
Mapping & understanding the homecare population, providers, and workforce.	2	4	5	2
Public sector funding of homecare.	0	3	3	2
Homecare compared to other care options.	2	6	6	1
Timely engagement with homecare.	0	1	0	3
Navigating & decision-making about homecare.	1	2	2	4
Homecare as a preventive health intervention.	2	2	5	2
Understanding and comparing the different models of homecare …….	0	1	2	1
Integrating an enabling approach into homecare.	0	0	2	3
Understanding homecare as a relationship-based intervention.	0	0	3	5
Homecare as a social intervention.	2	0	2	6
Joint working between homecare and health care services.	0	1	0	1
Family involvement in home care.	1	0	1	2
Housing and homecare.	2	3	7	4
Workforce: recruitment & retention.	0	1	2	0
Workforce: supervision, support and training.	1	0	0	3
Technology: supporting the delivery of care.	3	8	7	3
Technology: meeting care needs.	3	3	4	4
Managing complaints about homecare.	1	3	1	10
Person-centred needs assessment.	0	0	1	3
