# Supplementary material for: Research priorities for homecare for older people: A UK multi‐stakeholder consultation
Source: Health Soc Care Community. 2022 Sep 22;30(6):e5647–60. doi: 10.1111/hsc.13991 (PMC10087309; doi:10.1111/hsc.13991)
Supplement: Supplementary file 5 — Data S5 [file HSC-30-e5647-s003.pdf]

Supporting Information 5. Systematic reviews

Topic area	Citation (review protocol or output paper)	Review title	Status	Date range of searches	Number of studies & study participants
Mapping & understanding the homecare population, providers, and workforce.	Lucien, Zwakhalen, Morenon, & Hahn (2020)	Violence toward formal and informal caregivers and its consequences in the homecare setting: a protocol for systematic mixed studies review.	Ongoing (Registered Oct 2020)	Review on-going	n/a
Mapping & understanding the homecare population, providers, and workforce.	Backhouse, Ruston, Killett, & Mioshi (2021)	What do we know about safety, safeguarding and risk mitigation practices in paid homecare for people with dementia? A systematic review.	Ongoing (Registered Apr 2021)	Review on-going	n/a
Homecare compared to other care options.	Ogwu, Carey, Taylor, Alam, & Osborne (2020)	Homecare vs Residential Aged Care: A Systematic Review of Cost-Effectiveness and Quality of Life.	Ongoing (Registered Jul 2020)	Review on-going	n/a
Homecare compared to other care options.	Young et al. (2017)	Home or foster home care versus institutional long-term care for functionally dependent older people	Published 2017	Database inception - Nov 2015	10 studies; 16,337 participants
Homecare compared to other care options.	Montgomery, Mayo-Wilson, Dennis, & Mayo-Wilson (2008)	Personal assistance for older adults (65+) without dementia	Published 2008	1980 - Jun 2005	4 studies; 1642 participants

Homecare as a preventive health intervention.	Spiers et al. (2019)	Impact of social care supply on healthcare utilisation by older adults: a systematic review and meta-analysis	Published 2019	2000 - May 2018	12 studies, of which **3** evaluated homecare in terms of health outcomes. All homecare studies included in meta-analysis
Homecare as a preventive health intervention.	Dawson et al. (2020)	Does health and social care provision for the community dwelling older population help to reduce unplanned secondary care, support timely discharge and improve patient well-being? A mixed method meta-review of systematic reviews.	Published 2020	Jan 2013 - Mar 2018	Meta review of 71 systematic reviews, of which **4** concerned impacts of homecare on health outcomes.
Integrating an enabling approach into homecare.	Bennett & Hodge (2021)	How effective are the occupational therapy (OT) interventions, delivered during reablement homecare service?	Ongoing (Registered Feb 2021)	Review on-going	n/a
Integrating an enabling approach into homecare.	Whitehead, Worthington, Parry, Walker, & Drummond, (2015)	Interventions to reduce dependency in personal activities of daily living in community dwelling adults who use homecare services: a systematic review.	Published 2015	Database inception- Nov 2014	13 studies; 4975 participants
Integrating an enabling approach into homecare.	Crocker et al. (2021)	Community-based complex interventions to sustain independence in older people, stratified by frailty: a	Ongoing (Registered Dec 2019)	Review on-going	n/a

		systematic review and network meta-analysis.			
Integrating an enabling approach into homecare.	Cochrane et al. (2016)	Time-limited home-care reablement services for maintaining and improving the functional independence of older adults	Published (2016)	Database inception -June 2015	2 studies;<br>811 participants
Homecare as a social intervention.	McGill, Malden, Alex, et al. (2021)	Exploring the embedded networks of care for older adults: a systematic review.	Ongoing (Registered Jul 2021)	Review on-going	n/a
Workforce: supervision, support and training.	Cunningham, Cowie, Watchman, & Methven (2020)	Understanding the training and education needs of homecare workers supporting people with dementia and cancer: A systematic review of reviews	Published 2020	2010 to not stated	13 systematic reviews included
Technology: meeting care needs.	Malden et al. (2021)	Digital technologies to enable older people to remain in their living environments: an umbrella review.	Ongoing (Registered Oct 2021)	Review on-going	n/a
Technology: meeting care needs.	McGill, Malden, Frost, et al. (2021)	A systematic review of the facilitators and barriers to implementing new technologies to support paid and unpaid care for older adults.	Ongoing (Registered Jul 2021)	Review on-going	n/a
Technology: meeting care needs.	Szczepura et al. (2020)	Technology introduction in care environments: systematic review of living lab models in diverse care settings.	Ongoing (Registered Nov 2020)	Review on-going	n/a

References

Backhouse, T., Ruston, A., Killett, A., & Mioshi, E. (2021). What do we know about safety, safeguarding and risk mitigation practices in paid homecare for people with dementia? A systematic review. Available from 01/04/2021 PROSPERO Retrieved 10.12.2021, from PROSPERO CRD42021246621

Bennett, C., & Hodge, S. (2021). A systematic review of the effectiveness of occupational therapy interventions delivered in reablement homecare. Available from 14/02/2021 PROSPERO Retrieved 10.12.2021, from PROSPERO CRD42021237209

Cochrane, A., Furlong, M., McGilloway, S., Molloy, D., Stevenson, M., & Donnelly, M. (2016). Time-limited home-care reablement services for maintaining and improving the functional independence of older adults. *Cochrane Database of Systematic Reviews*(10). https://doi:http://dx.doi.org/10.1002/14651858.CD010825.pub2

Crocker, T. F., Clegg, A., Riley, R. D., Lam, N., Bajpai, R., Jordão, M., . . . Forster, A. (2021). Community-based complex interventions to sustain independence in older people, stratified by frailty: a protocol for a systematic review and network meta-analysis. *BMJ open, 11*(2), e045637. http://dx.doi.org/10.1136/bmjopen-2020-045637

Cunningham, N., Cowie, J., Watchman, K., & Methven, K. (2020). Understanding the training and education needs of homecare workers supporting people with dementia and cancer: A systematic review of reviews. *Dementia (London), 19*(8), 2780-2803. https://doi:10.1177/1471301219859781

Dawson, S., Kunonga, P., Beyer, F., Spiers, G., Booker, M., Ruth, M., . . . Salisbury, C. (2020). Does health and social care provision for the community dwelling older population help to reduce unplanned secondary care, support timely discharge and improve patient well-being? A mixed method meta-review of systematic reviews. *F1000Research, 9*(857). https://dx.doi.org/10.12688%2Ff1000research.25277.1

Lucien, B., Zwakhalen, S., Morenon, O., & Hahn, S. (2020). Violence toward formal and informal caregivers and its consequences in the homecare setting: a protocol for systematic mixed studies review. Available from 17/09/2020 PROSPERO Retrieved 10.12.2021, from PROSPERO CRD42020209836

Malden, S., McGill, K., Shenkin, S., Hanratty, B., Arslan, T., Fleuriot, J., . . . Guthrie, B. (2021). Digital technologies to enable older people to remain in their living environments: an umbrella review. Available from 05/10/2021 PROSPERO Retrieved 11.12.2021, from PROSPERO CRD42021283484

McGill, K., Malden, S., Alex, B., Aujila, N., Kaner, E., Lewis, S., . . . Wilson, C. (2021). Exploring the embedded networks of care for older adults: a systematic review. Available from 09/07/2021 PROSPERO Retrieved 11.12.2021, from PROSPERO CRD42021266849

McGill, K., Malden, S., Frost, H., Lewis, S., Mercer, S., O'Donnell, A., . . . Wilson, C. (2021). A systematic review of the facilitators and barriers to implementing new technologies to support paid and unpaid care for older adults. Available from 08/07/2021 PROSPERO Retrieved 11.12.2021, from PROSPERO CRD42021266656

Montgomery, P., Mayo-Wilson, E., Dennis, J. A., & Mayo-Wilson, E. (2008). Personal assistance for older adults (65+) without dementia. *Cochrane Database of Systematic Reviews*(10). https://doi.org/10.1002/14651858.CD006855.pub2

Ogwu, E., Carey, M., Taylor, M., Alam, K., & Osborne, S. (2020). Homecare vs Residential Aged Care: A Systematic Review of Cost-Effectiveness and Quality of Life (Protocol). Available from 08/04/2020 PROSPERO Retrieved 10.12.2021, from PROSPERO CRD42020178886

Spiers, G., Matthews, F. E., Moffatt, S., Barker, R. O., Jarvis, H., Stow, D., . . . Hanratty, B. (2019). Impact of social care supply on healthcare utilisation by older adults: a systematic review and meta-analysis. *Age Ageing, 48*(1), 57-66. https://doi:10.1093/ageing/afy147

Szczepura, A., Nomura, T., Wild, D., Ouillon, S., Bark, C., Collinson, M., . . . Loizou, M. (2020). Technology introduction in care environments: systematic review of living lab models in diverse care settings. Available from 31/10/2020 PROSPERO Retrieved 11.12.2021, from PROSPERO CRD42020218362

Whitehead, P. J., Worthington, E. J., Parry, R. H., Walker, M. F., & Drummond, A. E. (2015). Interventions to reduce dependency in personal activities of daily living in community dwelling adults who use homecare services: a systematic review. *Clin Rehabil, 29*(11), 1064-1076. https://doi:10.1177/0269215514564894

Young, C., Hall, A. M., Gonçalves-Bradley, D. C., Quinn, T. J., Hooft, L., van Munster, B. C., & Stott, D. J. (2017). Home or foster home care versus institutional long-term care for functionally dependent older people. *Cochrane Database of Systematic Reviews*(4). https://doi:10.1002/14651858.CD009844.pub2
