# Supplementary material for: Research priorities for homecare for older people: A UK multi‐stakeholder consultation
Source: Health Soc Care Community. 2022 Sep 22;30(6):e5647–60. doi: 10.1111/hsc.13991 (PMC10087309; doi:10.1111/hsc.13991)
Supplement: Supplementary file 6 — Data S6 [file HSC-30-e5647-s002.pdf]

Supplementary Information 6. UK Based Research Studies.

Topic area	Chief Investigator and output paper(s)	Study title and project page	Status	Research funder	Objective/ focus of study
Mapping & understanding the homecare population, providers, and workforce.	Michael Robling (Lugg-Widger et al., 2020)	Establishing the impact of COVID-19 on the health outcomes of domiciliary care workers in Wales using routine data<br><br>(UKRI, 2020)	Ongoing. Due to complete 2022	ESRC	Impact of Covid-19 on homecare workers in Wales; enabling extrapolation to other UK populations.
Navigating & decision-making about homecare	Caroline Glendinning (Rabiee & Glendinning, 2014) (Rabiee, Baxter, & Glendinning, 2016)	Personalisation of home care for older people using managed personal budgets<br><br>(NIHR SSCR, 2013)	Completed 2013	NIHR SSCR	Exploration of factors affecting the delivery of personalised home care to older people who opt for council-managed personal budgets rather than cash direct payments.
Navigating & decision-making about homecare	Frances Bunn (Bunn et al., 2018)	Supporting shared decision making for older people with multiple health and social care needs: a realist synthesis to inform emerging models of health and social care	Completed 2017	NIHR HSDR	Models of shared decision-making for older people with multiple health and care needs; how they might be applied to integrated care models.

		(NIHR HSDR, 2017b)			
Navigating & decision-making about homecare	Mark Wilberforce (Wilberforce et al., 2020)	Self-funders' preferences for support in navigating community social care.<br><br>(NIHR SSCR, 2020)	Ongoing. Due to complete 2022	NIHR SSCR	What self-funders most value when seeking, choosing and arranging their social care, and how different groups of self-funders make decisions. How to improve support for self-funders in taking social care decisions.
Understanding and comparing the different models of homecare	Caroline Needham (Needham et al., 2015)	Does Smaller mean Better? Evaluating Micro-Enterprises in Adult Social Care<br><br>(UKRI, 2015)	Completed 2015	ESRC	User experience of micro-enterprise social care providers (including homecare) compared with larger organisations.
Understanding and comparing the different models of homecare.	David Challis<br><br>(Jasper et al., 2019)	Commissioning Home Care for Older People.<br><br>(NIHR SSCR, 2019)	Completed 2019	NIHR SSCR	The appraisal of the components of the commissioning process and exploration of current arrangements.
Integrating an enabling approach into homecare.	Bryony Beresford (Beresford et al., 2019)	Models Of Reablement Evaluation: a mixed methods evaluation of a complex intervention (The MORE project)<br><br>(NIHR HSDR, 2017a)	Completed 2019	NIHR HSDR	Effectiveness of different delivery models of reablement services.
Understanding homecare as a	Eleanor Johnson	COVID-19, closeness, and care: The changing provision of direct care for older people	Ongoing. Due to	NIHR SSCR	The impact of Covid-19 on caregiving relationships, including the close and intimate

relationship-based intervention.		in residential and home care settings.<br><br>(NIHR SSCR, 2021)	complete 2022		aspects of care giving and the meaning and value care givers find in their labour.
Housing and homecare.	Naomi Kingsley - Project Manager	Healthy Homes, Healthy Lives<br><br>(UKRI, 2021)	Ongoing. Due to completed 2024	Innovate UK	Ageing in place services development project aimed at older homeowners. Research element via end user surveys and interviews conducted by the London School of Economics.
Workforce: supervision, support and training	Justine Schneider (Turner et al., 2018) (Schneider et al., 2019)	Defining quality home care for people with dementia: A mixed methods study.<br><br>(NIHR SSCR, 2018)	Completed 2018	NIHR SSCR	Examine home care for people with dementia so that its function in community support can be described fully, with a view to service development and effective commissioning.
**Key:**	NIHR = National Institute for Health Research.			SSCR = School for Social Care Research	
	ESRC = Economic and Social Research Council.			HSDR = Health and Social Care Delivery Research	

References

Beresford, B., Mann, R., Parker, G., Kanaan, M., Neves De Faria, R., I, Rabiee, P., . . . Duarte, A. (2019). Reablement services for people at risk of needing social care: the MoRe mixed-methods evaluation. *Health services and delivery research*, 1-254. https://eprints.whiterose.ac.uk/145437/1/Reablement_services_for_people_at_risk_of_needing_social_care_the_MoRe_mixed_methods_evaluation.pdf

Bunn, F., Goodman, C., Russell, B., Wilson, P., Manthorpe, J., Rait, G., . . . Durand, M.-A. (2018). Supporting shared decision making for older people with multiple health and social care needs: a realist synthesis. *BMC geriatrics, 18*(1), 1-16. https://doi.org/10.1186/s12877-018-0853-9

Jasper, R., Hughes, J., Roberts, A., Chester, H., Davies, S., & Challis, D. (2019). Commissioning home care for older people: Scoping the evidence. *Journal of Long-Term Care, 2019*, 176-193. https://doi.org/10.31389/jltc.9

Lugg-Widger, F., Cannings-John, R., Akbari, A., Brookes-Howell, L., Hood, K., John, A., . . . Thomas, D. (2020). Establishing the impact of COVID-19 on the health outcomes of domiciliary care workers in Wales using routine data: a protocol for the OSCAR study. *International Journal of Population Data Science, 5*(4). https://dx.doi.org/10.23889%2Fijpds.v5i4.1656

Needham, C., Allen, K., Hall, K., Mckay,S., Glasby, J. Carr, S., Littlechild, R. & Carr, S. (2015). *Micro-enterprises: small enough to care?* University of Birmingham Retrieved from: https://pureoai.bham.ac.uk/ws/files/26718275/Needham_2015_MicroEnterprise_FullReport.pdf?_ga=2.116367364.38685772.1641913404-1752348093.1629737199

NIHR HSDR. (2017a). Models Of Reablement Evaluation: a mixed methods evaluation of a complex intervention (The MORE project). https://fundingawards.nihr.ac.uk/award/13/01/17.

NIHR HSDR. (2017b). Supporting shared decision making for older people with multiple health and social care needs: a realist synthesis to inform emerging models of health and social care. https://fundingawards.nihr.ac.uk/award/15/77/25.

NIHR SSCR. (2013). Personalisation of home care for older people using managed personal budgets. https://www.sscr.nihr.ac.uk/projects/p11/

NIHR SSCR. (2018). Defining quality home care for people with dementia: A mixed-methods study. https://www.sscr.nihr.ac.uk/projects/p67/

NIHR SSCR. (2019). Commissioning home care for older people. https://www.sscr.nihr.ac.uk/projects/p96/

NIHR SSCR. (2020). Self funders' preferences for support in navigating community social care. https://www.sscr.nihr.ac.uk/projects/p140/

NIHR SSCR. (2021). COVID-19, closeness, and care: The changing provision of direct care for older people in residential and home care settings. https://www.sscr.nihr.ac.uk/projects/p186/

Rabiee, P., Baxter, K., & Glendinning, C. (2016). Supporting choice: Support planning, older people and managed personal budgets. *Journal of Social Work, 16*(4), 453-469. https://doi.org/10.1177%2F1468017315581529

Rabiee, P., & Glendinning, C. (2014). Choice and control for older people using home care services: how far have council-managed personal budgets helped? *Quality in Ageing and Older Adults*. https://doi.org/10.1108/QAOA-04-2014-0007

Schneider, J., Pollock, K., Wilkinson, S., Perry-Young, L., Travers, C., & Turner, N. (2019). The subjective world of home care workers in dementia: An “order of worth” analysis. *Home Health Care Services Quarterly, 38*(2), 96-109. https://doi.org/10.1080/01621424.2019.1578715

Turner, N., Schneider, J., Pollock, K., Travers, C., Perry-Young, L., & Wilkinson, S. (2018). 'Going the extra mile' for older people with dementia: Exploring the voluntary labour of homecare workers. *Dementia (London)*, 1471301218817616. https://doi.org/10.1177%2F1471301218817616

UKRI. (2015). Does Smaller mean Better? Evaluating Micro-Enterprises in Adult Social Care. https://gtr.ukri.org/projects?ref=ES%2FK002317%2F1

UKRI. (2020). Establishing the impact of COVID-19 on the health of domiciliary care workers in Wales: developing a model for UK service planning and carer support. https://gtr.ukri.org/projects?ref=ES%2FV015206%2F1

UKRI. (2021). Healthy Homes, Healthy Lives. https://gtr.ukri.org/projects?ref=99226

Wilberforce, M., Baxter, K., Birks, Y., Tonks, S., Jasper, R., Wright, S., & Morfitt, R. (2020). Self-funders' preferences for support in navigating community social care (Outline of research study in progess.). Retrieved 15th December 2021, from NIHR https://www.sscr.nihr.ac.uk/projects/p140/
